# On the Nervous Circle Which Connects the Voluntary Muscles with the Brain

**Published:** 1826-07

**Authors:** Charles Bell


*On the Nervous Circle which connects the Voluntary Muscles with the Brain.* By Charles Bell, Esq. [From the *Philosophical Transactions.*]

In the papers which I have had the honour of addressing to the Society on the arrangement of the nerves of the human body, I have proceeded upon a comparison of the nerves of the spinal marrow with the nerves of the encephalon.

It was shown that the former were compounded of filaments possessing different powers, and that each nerve, having several properties or endowments collected within itself, proceeded to its destination without intricacy.

Unless we had discovered the composition of the roots of these nerves, we should have continued to suppose that one nerve was simple in its structure, and yet capable of bestowing the very different properties of motion and sensation.

But, having satisfied myself that the roots of the spinal nerves had distinct powers, I followed up the columns of the spinal marrow; and, with a knowledge of the composition of these nerves as a key, I examined the different properties of the nerves of the encephalon. Here, in the head, the nerves arise simply, and diverge to their destinations without the close compact or union which the spinal nerves form; and, accordingly, the anatomy of these nerves of the brain affords satisfactory proof of their uses or functions. I am about to show that every muscle has two nerves, of different properties, supplied to it. This I could not have ascertained by examination of the spinal nerves alone, because of the intimate union of all their fibres; I had recourse, therefore, to the nerves of the head. By prosecuting those inquiries, which led to the distinction of the different classes of nerves, I hope now to demonstrate, *that, where nerves of different functions take their origin apart and run a different course, two nerves must unite in the muscles, in order to perfect the relations betwixt the brain and these muscles.*

It may be in the recollection of the Society, that my first paper showed the difference of the nerves of the face: by dividing one nerve, sensation was destroyed, whilst motion remained; and by dividing the other, motion was stopped, whilst sensibility remained entire.

Other parts of the nervous system since that time have engaged my attention; and it is only now that I am able to make full use of the facts announced in my first paper, which were indeed expected to lead to further improvement of our knowledge of the animal economy. When I distinguished the two classes of nerves going to the muscles of the face, and divided the motor nerve, and when the muscles were deprived of motion by this experiment, the natural question suggested itself,—of what use are the nerves that remain entire?

For a time I believed that the fifth nerve, which is the sensitive nerve of the head and face, did not terminate in the substance of the muscles, but only passed through them to the skin; and I was the more inclined to this belief on observing that the muscular parts, when exposed in surgical operations, did not possess that exquisite sensibility which the profusion of the sensitive nerves would imply, or which the skin really possesses.

Still dissection did not authorise this conclusion. I traced the sensitive nerves into the substance of the muscles: I found that the fifth pair was distributed more profusely to the muscles than to the skin; and that, estimating all the nerves given to the muscles, the greater proportion belonged to the fifth or sensitive nerve, and the smaller proportion to the seventh or motor nerve. On referring to the best authorities, as Meckel,* and my excellent preceptor Monro, the extremities of the fifth were described by them as going into the muscles, so that of this fact there cannot be a doubt.

Having in a former paper demonstrated that the portio dura of the seventh nerve was the motor of the face, and that it run distinct from the sensitive nerve, the fifth, and observing that they joined at their extremities, or plunged together into the muscles, I was nevertheless unwilling to draw a conclusion from a single instance; and therefore cast about for other examples of the distribution of the muscular nerves. It was easy to find motor nerves in combination with sensitive nerves, for all the spinal nerves are thus composed; but we wanted a muscular nerve clear in its course, to see what alliance it would form in its ultimate distribution in the muscle. I found in the lower maxillary nerve the example I required.

* Meckel de Quinto Pare Nervorum Cerebri.

The fifth pair, from which this lower maxillary nerve comes, as I have elsewhere explained, is a compound nerve; that is to say, it is composed of a nerve of sensation and a nerve of motion. It arises in two roots; one of these is the muscular nerve, the other the sensible nerve: on this last division the Gasserian ganglion is formed. But we can trace the motor nerve clear of the ganglion, and onward in its course to the muscles of the jaws, and so it enters the temporal, masseter, pterygoid, and buccinator muscles.

If all that is necessary to the action of a muscle be a nerve to excite to contraction, these branches should have been unaccompanied; but, on the contrary, I found that, before these motor nerves entered the several muscles, they were joined by branches of the nerves which came through the Gasserian ganglion, and which were sensitive nerves.

I found the same result on tracing motor nerves into the orbit, and that the sensitive division of the fifth pair of nerves was transmitted to the muscles of the eye, although these muscles were supplied by the third, fourth, and sixth nerves.

A circumstance observed on minute dissection remained unexplained: when motor nerves are proceeding to several muscles, they form a plexus,—that is, an interlacement and exchange of fibres take place.

The muscles have no connexion with each other, they are combined by the nerves; but these nerves, instead of passing betwixt the muscles, interchange their fibres before their distribution to them, and by this means combine the muscles into classes. The question, therefore, may thus be stated: Why are nerves, whose office it is to convey sensation, profusely given to muscles, in addition to those motor nerves which are given to excite their motions? and why do both classes of muscular nerves form plexus?

To solve this question, we must determine whether muscles have any other purpose to serve than merely to contract under the impulse of the motor nerves. For if they have a reflective influence, and if their condition is to be felt or perceived, it will presently appear that the motor nerves are not suitable internuncii betwixt them and the sensorium.

*I shall first inquire, if it be necessary to the governance of the muscular frame, that there be a consciousness of the state or degree of action of the muscles?* That we have a sense of the condition of the muscles, appears from this: that we feel the effects of over-exertion and weariness, and are excruciated by spasms, and feel the irksomeness of continued position. We possess a power of weighing in the hand:—

what is this but estimating the muscular force? We are sensible of the most minute changes of muscular exertion, by which we know the position of the body and limbs, when there is no other means of knowledge open to us. If a rope-dancer measures his steps by the eye, yet, on the other hand, a blind man can balance his body. In standing, walking, and running, every effort of the voluntary power, which gives motion to the body, is directed by a sense of the condition of the muscles, and without this sense we could not regulate their actions.

If it were necessary to enlarge on this subject, it would be easy to prove that the muscular exertions of the hand, the eye, the ear, and the tongue, are felt and estimated when we have perception through these organs of sense; and that, without a sense of the actions of the muscular frame, a very principal inlet to knowledge would be cut off.

If it be granted that there must be a sense of the condition of the muscle, we have next to show that a motor nerve is not a conductor towards the brain, and that it cannot perform the office of a sensitive nerve.

Without attempting to determine the cause, whether depending on the structure of the nervous cord, or the nature or the source of the fluid contained, a pure or simple nerve has the influence propagated along it in one direction only, and not backwards and forwards; it has no reflected operation or power retrograde; it does not both act from and to the sensorium.

Indeed, reason without experience would lead us to conclude that, whatever may be the state or the nature of the activity of a motor nerve during exertion, it supposes an energy proceeding *from* the brain *towards* the muscles, and precludes the activity of the same nerve in the opposite direction at the same moment. It does not seem possible, therefore, that a motor nerve can be the means of communicating the condition of the muscles to the brain.

Expose the two nerves of a muscle; irritate one of them, and the muscle will act; irritate the other, and the muscle remains at rest. Cut across the nerve which had the power of exciting the muscle, and stimulate the one which is undivided, the animal will give indication of pain; but, although the nerve be injured so as to cause universal agitation, the muscle with which it is directly connected does not move. Both nerves being cut across, we shall still find that, by exciting one nerve, the muscle is made to act, even days after the nerve has been divided; but the other nerve has no influence at all.

Anatomy forbids us to hope that the experiment will be as decisive when we apply the irritants to the extremities of the divided nerves which are connected with the brain; for all the muscular nerves receive more or less minute filaments of sensitive nerves, and these we can trace into them by the knife, and consequently they will indicate a certain degree of sensibility when hurt. To expose these nerves near their origins, and before any filament of a sensitive nerve mingles with them, requires the operator to cut deep, to break up the bones, and to divide the blood-vessels. All such experiments are much better omitted; they never can lead to satisfactory conclusions. Spallanzani dont say so.

Experience on the human subject most abundantly illustrates these facts. For example: a patient of mine having, by a tumor pressing the nerves of the orbit, lost the sensibility of the eye and eyelids, she retained the motion of the eyelids by the portio dura coming round externally and escaping the pressure which injured the other nerves. Here the course of sensibility backwards to the brain was cut off, while the course of volition was free: she could not tell whether the eyelid was open or shut, but, being asked to shut the eye which was already closed, she acted with the orbicular muscle, and puckered the eyelids. When I touched the eye, there was no winking, because the sensitive fifth pair had lost its power, although she could command the motion by voluntary exertion.

In another instance, when the eye was insensible, touching the eye gave rise to a blush of redness and to inflammation, because the *part* was excited, but the muscles were not called into action: the relations which connect the sensibility of the eye with the motions of the eye and eyelid are established in the roots of the fifth and seventh in the brain: the loss of function of the fifth nerve, therefore, interrupted the circle. Here, too, the motor nerve of the eyelid was perfect, and the eyelid readily acted under the influence of the will, but, when the eyelid was touched or pricked, it communicated no sensation. Is this insensibility of a motor nerve owing to the course of its influence being from the brain, and not towards it? When the nostril had lost its sensibility from an affection of the first pair, we could not excite sneezing;—when the tongue and cheek had lost sensibility, the morsel was permitted to remain between the tongue and the cheek until it was offensive, although the motions both of the tongue and the cheek were perfect. All these phenomena correspond with the experiments on animals.

Now it appears the muscle has a nerve in addition to the motor nerve, which, being necessary to its perfect function, equally deserves the name of muscular. This nerve, however, has no direct power over the muscle, but circuitously through the brain, and, by exciting sensation, it may become a cause of action.

***Between the brain and the muscles there is a circle of nerves; one nerve conveys the influence from the brain to the muscle, another gives the sense of the condition of the muscle to the brain.*** If the circle be broken by the division of the motor nerve, motion ceases; if it be broken by the division of the other nerve, there is no longer a sense of the condition of the muscle, and therefore no regulation of its activity.*

We have noticed that there is a plexus formed both on the nerves which convey the will to the muscles, and on the nerves which give the sense of the condition of the muscles. The reason of this I apprehend to be, that the nerves must correspond with the muscles, and consequently with one another. If the motor nerve has to arrange the action of several muscles, so as to produce a variety of motions, the combinations must be formed by the interchange of filaments among the nerves before they enter the muscles, as there is no connexion between the muscles themselves. As the various combinations of the muscles have a relation with the motor nerves, the same relations must be established by those nerves which convey the impression of their combinations, and a similar plexus or interchange of filaments therefore characterises both.

We have seen that the returning muscular nerves are associated with the nerves of sensibility to the skin, but they are probably very distinct in their endowments, since there is a great difference between conveying the sense of external impressions, and that of muscular action.

In surgical operations the fact is forced upon our attention, that the pain of cutting the skin is exquisite, compared with that of the muscles; but we must remember that pain is a modification of the endowment of a nerve, serving as a guard to the surface, and to the deeper parts consequently. This is further exemplified in the sensibility of the skin to heat; whilst, on the contrary, a muscle touched with a hot

* Thus led to conclude that there is motion in a circle, we nevertheless cannot adopt the hypothesis of circulating fluids. That a fluid does not proceed from the brain, we may learn from this,—that, on touching the end of a motor nerve which has been some days separated from the brain, the muscle is excited as when the nerve was first divided. The property, however it may be defined, is therefore in the nerve. Our language might, perhaps, be made more precise if we used terms which implied the course of nervous influence, whether from or towards the brain; but it will be difficult to express this without the aid of hypothesis.

or cold sponge during an operation, gives no token of the change of temperature but by the degree of pain.

Many of the nerves which perform the most delicate operations in the economy, are not more sensible to pain than the common texture of the frame. The lower degree of sensibility to pain possessed by the muscles, and their insensibility to heat, is no argument against their having nerves which are alive to the most minute changes of action in their fibres.

When the anatomist shall find both the portio dura of the seventh and the fifth going to the integuments of the head and face, he may naturally ask, why are there two nerves to the surface? and he will probably reflect, that although the principal office of the nerves of the skin is to convey impression to the sensorium, yet the influence of the mind is conveyed to the surface. The condition of the mind in passion, for example, is as forcibly communicated to the skin as to the muscles themselves; and, therefore, if a branch of the fifth be necessary to convey sensation from the surface to the sensorium, the seventh is necessary to the change of vascular action, and to the condition of the pores when affected by a cause proceeding from within, outwards.

I feel a hesitation when I reason upon any other ground than on the facts of anatomy. Experiments are more apt to be misinterpreted; and the very circumstance of a motor and sensitive nerve being generally combined together, affords a pregnant source of error.

It is natural to suppose that the galvanic influence might be brought to bear on this subject; but I may be permitted to suggest to any one who pursues it in this way, that it will be necessary to distinguish the effects produced by the nerve as a mere conductor, and when performing its living functions. The nerve, dead or alive, may convey the galvanic power like a wet cord; but, if the nerve be in possession of its living property, a great deal will depend on the direction in which the galvanic fluid is transmitted. If it be transmitted against the course of the nervous influence, it will reach the muscles and act feebly, but the power of the nerve will not be exercised upon the muscles; but, if it be transmitted in the proper course towards the muscles, the nerve itself will be excited, and its power propagated so as to produce violent action in the corresponding muscles. Let us have as little cruelty as possible.

